# Access, Readiness and Willingness to Engage in Allied Health Telerehabilitation Services for Adults: Does Cultural and Linguistic Diversity Make a Difference?

**DOI:** 10.3390/healthcare12111141

**Published:** 2024-06-04

**Authors:** Clarice Y. Tang, Andisheh Bastani, Balwinder Sidhu, Golsa Saberi, Elise Baker

**Affiliations:** 1 School of Health Sciences, Western Sydney University, Campbelltown, NSW 2560, Australia; elise.baker@westernsydney.edu.au; 2 Allied Health, South Western Sydney Local Health District, Liverpool, NSW 2170, Australia; 3 College of Sport, Health and Engineering, Victoria University, Melbourne, VIC 3011, Australia; 4 School of Health Sciences, Swinburne University of Technology, Hawthorn, VIC 3122, Australia; 5 Multicultural Services, South Western Sydney Local Health District, Liverpool, NSW 2170, Australia; 6 Ingham Institute for Applied Medical Research, Liverpool, NSW 2170, Australia

**Keywords:** telerehabilitation, allied health, culturally and linguistically diverse, access, readiness, willingness

## Abstract

Telerehabilitation is an appealing service delivery option for optimising recovery. Internationally, the equity of telerehabilitation services for people from culturally and linguistically diverse (CALD) backgrounds has been questioned. Using a 31-item survey, our study explored the access, readiness and willingness of 260 patients receiving allied health services from a large tertiary health service located in Sydney, Australia, to use telerehabilitation for adults. Overall, 72% patients reported having access to technology, 38% met our readiness criteria and 53% reported willingness to engage in telerehabilitation. There were no differences in access, readiness and willingness to engage in telerehabilitation between patients from CALD and non-CALD backgrounds. Age was the only factor that influenced access (OR = 0.94, 95% CI 0.90 to 0.97), readiness (OR = 0.95, 95% CI 0.92 to 0.98) and willingness (OR = 0.97, 95% CI 0.95 to 1.00) to engage in telerehabilitation. Past experience of telerehabilitation was related to willingness (OR = 2.73, 95% CI 1.55–4.79) but not access (OR = 1.79, 95% CI 0.87 to 3.68) or readiness (OR = 1.90, 95% CI 0.93 to 3.87). Our findings highlight the importance of ensuring positive patient experiences to promote ongoing willingness to use telerehabilitation. Efforts are needed to improve patients’ digital health literacy, especially patients from older age groups, to ensure equitable engagement in telerehabilitation services.

## 1. Introduction

Telerehabilitation has become an increasingly appealing option for the delivery of allied health rehabilitation services to optimise recovery [1,2]. This increased appeal has occurred particularly since COVID-19 [3]. Telerehabilitation is a type of telehealth focused on “the delivery of rehabilitation and habilitation services via a variety of ICT [information and communication technologies]”, with the range of services encompassing “evaluation, assessment, monitoring, prevention, intervention, supervision, education, consultation and coaching” [4]. Although telerehabilitation can involve a range of technologies (e.g., wearable sensors, telepresence robots) [5,6], the focus of our study is on the use of telephone and video teleconferencing for the synchronous delivery of adult outpatient allied health rehabilitation services including physiotherapy, occupational therapy, podiatry, dietetics and speech-language pathology.

Research comparing the effectiveness of telerehabilitation suggests that telerehabilitation can be equally as effective as in-person services [7,8,9,10]. Telerehabilitation has also been reported to promote higher rates of attendance [11] and reduce costs for patients compared to in-person services [12,13]. Despite the equivalence and mounting benefits of telerehabilitation, equity in the engagement in telerehabilitation poses a challenge. One group where the equity of use has been questioned is for patients from culturally and linguistically diverse (CALD) backgrounds [14,15,16]. For the purposes of this paper, a patient is considered CALD if they are “born in non-English-speaking countries and/or their main language spoken at home is not English” [17]. It is a term used in Australian health care settings synonymous to cultural and ethnic minorities [17].

The potential inequities in telerehabilitation for patients from CALD communities are evident in two contexts. First, patients from CALD communities have been underrepresented in telerehabilitation research because they have either been explicitly excluded from participating because they could not understand or use spoken English (e.g., Seidman et al., (2017)) or passively excluded because strategies to include CALD patients have not been reported. Second, when the demographic reach of telehealth services has been studied, patients from CALD backgrounds have been reported to be less likely to engage in telehealth services compared to patients from non-CALD backgrounds [14,18,19]. This research has primarily been on telehealth for acute and non-acute medical services in the USA [14] rather than telerehabilitation for allied health services in Australia. Given that people from CALD communities in Australia have been reported to face inequitable challenges in accessing health care services more broadly [20], it could be presumed they also face challenges in using telerehabilitation services.

One obvious barrier to engagement with telerehabilitation is having or being able to access telephone and/or video teleconference devices (e.g., a smart phone, computer, or tablet) with affordable Internet access and data [21]. Having access, however, does not guarantee that people will engage in the use of telerehabilitation. Across the extant literature on the access to and use of telehealth, researchers have explored two related concepts: readiness to engage in telehealth [22,23] and willingness to use telerehabilitation [24,25].

Readiness is a multidimensional construct capturing both access and the skills or abilities related to using a computer and the Internet [23]. Across the research on telehealth readiness, the repeated finding has been that older adults have lower telehealth readiness, despite having access to relevant technologies [23,26] (Hall Dykgraaf et al., 2022; van Houwelingen et al., 2018). This finding aligns with reports that older adults have lower digital literacy [26,27]. Compared to age, the influence of CALD background on readiness for telerehabilitation has been understudied.

As a complement to readiness, willingness refers to a patient’s desire to try telehealth as a model of service delivery to address their health condition [24,25]. Although it could be speculated that patients from CALD communities may have less access, and may have poorer readiness (based on poorer digital literacy), the limited research on the willingness of patients from CALD communities to engage in allied health telerehabilitation services has revealed diverse views [15]. Specifically, in a mixed-methods study, Brady and colleagues (2023) explored adults’ perspectives on telehealth, including patients from CALD backgrounds and their health professionals [15]. Some patients’ comments during semi-structured interviews suggested that some were not willing to use telehealth, while others were willing.

If we are to better understand how allied health telerehabilitation might be used to optimise rehabilitation outcomes for patients from CALD backgrounds, there is a need to better understand their self-reported access, readiness and willingness to engage in telerehabilitation and explore the demographic factors underlying each of these three concepts. To ensure that the relevant issues around access, readiness and willingness are identified, it would be important to contextualise the issues within the context of the broader community and compare views about access, readiness and willingness with patients from non-CALD backgrounds. Therefore, the primary aim of our study was to describe the access to telerehabilitation among adults from CALD and non-CALD backgrounds attending allied health outpatient services, explore their readiness to engage in telerehabilitation and determine their willingness to accept the use of telerehabilitation.

Our secondary aims were to (a) determine if there were any significant differences between CALD and non-CALD groups in terms of access, readiness and willingness to use telerehabilitation for allied health services and (b) investigate demographic factors influencing access, readiness and willingness to engage in telerehabilitation allied health outpatient services among patients from CALD and non-CALD backgrounds.

## 2. Materials and Method

A cross-sectional survey was conducted between January 2022 to February 2023 at a health service that provides care for people residing in the Greater Western Sydney region, home to one of the most culturally diverse communities (130 different cultural groups) in Sydney [28]. People residing in this area are also likely to be from a more socio-economically disadvantaged environment as compared to the general population in Sydney [29]. The SocioEconomic Indexes for Areas (SEIFA) of Greater Western Sydney is 967 (Australian Bureau of Statistics, 2021). This study received ethics approval from the local Human Research Ethics Committee.

A convenience sampling strategy was used to recruit patients who were attending outpatient allied health appointments with the health service. Participants included in the survey needed to be adults over the age of 18, attending outpatient allied health appointments at the health service and have sufficient cognition to participate in the survey as determined by their medical history. Participants also needed to be able to comprehend languages including English, Simplified Mandarin, Traditional Mandarin, Vietnamese and Arabic, the top four languages apart from English used at home across the Greater Western Sydney region (ABS, 2021). Patients who were unable to read and comprehend the selected languages as mentioned above were excluded. Additionally, patients who were currently participating in other research projects were excluded to avoid being over-burdened with the completion of the survey.

Potential participants were first approached by a member of the research team in the waiting room, who was not directly involved in the patients’ care. The member of the research team explained the purpose of the study. Based on the participants’ preferences, they would be provided either with a QR code that was linked to the study-specific survey or a hard copy of the survey in their preferred language. For participants who preferred to have someone reading the questions out to them, the member of the research team who was fluent in the selected language read the questions out for them but did not assist with the interpretation of questions. Participants also had the option of completing the survey at home. In this instance, participants were provided with a pre-paid envelope to facilitate the return of surveys.

The study-specific survey consisted of questions including demographic characteristics, access to technology, readiness to engage in telerehabilitation and their willingness to consider the use of technology (Supplementary Materials). Demographic characteristics such as age, sex, country of birth, perceived cultural identity, religion, socioeconomic status, home environment and level of education were captured using questions identical to the 2021 Australian Census [30]. The questions in the study were largely adapted from a previous study conducted by Seidman and colleagues (2017) [25], who looked at the extent of willingness to use telerehabilitation among people with chronic respiratory diseases. For the purpose of this study, readiness to use telerehabilitation was defined as a combination of having access to a smartphone, computer and/or the Internet and having a self-perceived data literacy rating of good or above. The survey was first piloted with a group of clinicians working at two local hospitals in the region to ensure that the questions asked were appropriately phrased for the patients. Questions were adjusted to ensure that they had a Flesch Reading score of between 90 and 100, indicating that these questions were considered to be easily understandable by an average 5th grader. The backward–forward translation method was also used to translate the survey into Vietnamese, Traditional Mandarin, Simplified Mandarin and Arabic.

Descriptive statistics were used to express categorical variables as counts and percentages (Table 1). The responses to access, employment and education were grouped into categories for analysis: (1) access, (2) no/limited access; (1) employed, (2) not employed/other and (3) retired; and (1) Year 8 or below, (2) Year 10 to diploma and (3) Bachelor and above, respectively. All analyses were conducted using SPSS statistics (version 29.0, IBM). Compliance with assumptions was checked using cross-tabulations and significant interactions were reported. A significance threshold of *p* < 0.05 was adopted in all analyses. A multivariable regression model was used to assess whether any of the demographic factors had an independent relationship in influencing the access, readiness and willingness to engage in telerehabilitation.

Considering that the health service provides services to approximately 70,000 patients annually (AIHW 2019), an estimated 196 participants were required to achieve results with a confidence level of 95% with a margin of error of 7%. Assuming a response rate of 80%, the study aimed to recruit a sample size of 245 participants [25].

## 3. Results

During the recruitment period from January 2022 to January 2023, 260 individuals participated in this study. Participants’ demographics and telerehabilitation service information are described in Table 1. Participants were, on average, 57 years old (SD = 20) and identified as female (64.2%; *n* = 167) (Table 1). More than half of the participants (*n* = 145) self-identified as being from a CALD background, while five did not provide any self-identification about their cultural and linguistic identity. Sixty-two percent of participants completed secondary school level education, with 40% living at home with their family. Only 30% of the participants were employed on either a full-time or part-time basis. Slightly over half of the participants (53.2%) had previous experience using telerehabilitation. A chi-square test of independence was performed to evaluate if there were significant between-group differences in demographic characteristics between CALD and non-CALD groups. Apart from education (χ^2^ (2, *N* = 255) = 12.28, *p* = 0.002), where there was a significant difference in the level of education between CALD and non-CALD groups, there were no other significant differences in demographic characteristics between the two groups. Multicollinearity between groups and education levels was not of concern (Tolerance = 1.00, VIF = 1.00).

### 3.1. Accessibility, Readiness and Willingness to Engage in Telerehabilitation

Almost three-quarters of the participants had access to technology, with a similar percentage of access between the CALD and non-CALD groups (Table 2). The vast majority of participants stated that they had access to smartphones. Less than half of the participants (40.7%) perceived themselves to have adequate computer/Internet skills, with similar percentages reported by both groups. In terms of readiness, only 38.4% of participants met the readiness criteria to use telerehabilitation, which included having access to technology and having a self-perceived rating score of at least a good level of computer/Internet skills. Over half of the participants (53%) stated that they would be willing to receive a telerehabilitation appointment in the future. There were no significant between-group differences in the level of access (χ^2^ (2, *N* = 250) = 0.48, *p* = 0.79), readiness (χ^2^ (1, *N* = 250) = 0.63, *p* = 0.43) and willingness (χ^2^ (1, *N* = 249) = 0.54, *p* = 0.46) to engage in telerehabilitation.

### 3.2. Regression Analysis for Access

A multivariable regression analysis was carried out to evaluate the relationship between access and demographic characteristics such as age, sex, self-identified cultural background, employment, education and past telerehabilitation experience. The results of the analysis is provided in Table 3.

Being of a younger age, being female and having a willingness to engage were independent factors that had a positive relationship in influencing the level of access to telerehabilitation (Table 3). Participants who were willing to engage in telerehabilitation had three times higher odds of having access to technology as compared to participants who were not willing to engage in telerehabilitation. All other factors such as self-identified cultural background, education, employment and past experiences of telerehabilitation did not have a significant relationship in influencing the level of access.

With regard to the readiness to engage in telerehabilitation, factors such as younger age, having higher levels of education and willingness to engage in telerehabilitation do have a significant relationship in influencing readiness to engage (Table 4). People who had a Bachelor’s degree and above had a 9.5 times higher odds of being ready to engage in telerehabilitation as compared to people with a year 8 or below level of education. Past experience of telerehabilitation did not have a significant relationship in influencing readiness to engage in telerehabilitation.

In terms of willingness to engage in telerehabilitation, only two factors had a relationship with the improvement in the willingness to engage in telerehabilitation (Table 5). Participants who had past telerehabilitation experience had a 2.7 times higher odds of being willing to engage in telerehabilitation as compared to participants who did not have past experiences with telerehabilitation. Age was another factor that influenced the willingness to engage in telerehabilitation; with every one year of age, there was a reduction in the odds of willingness to engage in telerehabilitation. Other demographic factors such as sex, self-identified cultural background, employment and education did not influence willingness to engage in telerehabilitation.

## 4. Discussion

Accessibility, level of education, employment status and willingness to engage in telerehabilitation are key elements that need to be considered when it comes to ascertaining the extent of engagement in telerehabilitation [22,31]. The findings from our study indicate that participants from a CALD background had similar access, readiness and willingness to engage in telerehabilitation as compared to people from a non-CALD background. The overall level of access, the level of digital literacy and the willingness to engage in telerehabilitation were low to moderate. While 72% of participants do have access to a device to carry out telerehabilitation, the overall access remained poorer than the national average in Australia [27]. According to the Australian Digital Inclusion Index report (2021), while the national access score increased from 70 in 2021 to 73 in 2023, this improvement in access was not evenly shared. Our results provide further evidence of a worrying gap in access to technology amongst people from socioeconomically disadvantaged backgrounds as compared to the general population.

The findings from our study do have significant implications to the future of telerehabilitation in Australia. Firstly, the results, contrasting with previous studies by [14,18,19], suggest that people from CALD backgrounds have similar access, readiness and willingness to engage in telerehabilitation as compared to people from non-CALD backgrounds. In support of our findings, there has been one study by Zhang and colleagues (2018) [32], who evaluated the technology use between people from CALD and non-CALD groups and found that even though usage pattern of technologies may be different, the level of access to technology was comparable between CALD and non-CALD groups. This raises the question as to why previous studies have found that people from CALD group were less likely to use telerehabilitation. It may be plausible that the contrasting results were due to the comparison of CALD groups with the general population rather than a sub-set of the population, which in this case focused on people from a lower socioeconomic status. This suggests that other underlying factors such as socioeconomic or educational levels, apart from being from a CALD background, could have a greater influence on engagement in telerehabilitation. Rather than suggesting that being from a CALD background has a direct contribution to the digital gap faced between people from CALD and non-CALD backgrounds, other factors such as age and lower educational status may play a bigger role in influencing readiness to engage in telerehabilitation. Health professionals should therefore provide an equal opportunity for all, regardless of CALD status to participate in telerehabilitation.

It is important to interpret our findings with caution. Firstly, it is vital to note that this study recruited people who are already currently accessing health services in the local area and did not capture people who are currently having difficulties accessing healthcare. Future research should recruit people who are currently having difficulties accessing healthcare to evaluate if being from a CALD background is an independent factor impacting on their ability or willingness to access and engage in telerehabilitation health services in the local area. Secondly, unlike previous studies such as [14,18,19], which reported on actual data usage or uptake of telerehabilitation or telehealth among people from CALD background, our study did not explore whether participants indeed engage in telerehabilitation. Therefore, it remains likely that people from a CALD background may not participate in telerehabilitation as often as people from a non-CALD background. Nonetheless, the findings from this study suggest that perhaps other factors such as patients’ self-belief in the efficacy of rehabilitation or their levels of health literacy may play a bigger role in influencing engagement in telerehabilitation among people from CALD backgrounds. Drawing parallels from recent studies evaluating the level of awareness of rehabilitation among people with chronic respiratory diseases, people from CALD backgrounds were less likely to be aware about rehabilitation [33,34], resulting in poorer referral rates and attendance in rehabilitation. It is also widely published that people from CALD backgrounds have poorer levels of health literacy as compared to people from non-CALD backgrounds (Khatri et al., 2022; Jessup et al., 2017) [20,35]. The lack of uptake of telerehabilitation among people from CALD backgrounds may also be due to the lack of understanding of the value of rehabilitation and low levels of health literacy. Further studies need to explore the impact of other factors beyond access, readiness and willingness to engage in telerehabilitation in the bid to explain why people from CALD backgrounds are less likely to engage in telerehabilitation.

Other factors impacting access to technology include age and sex. As shown in our study, the older the patient, the poorer the access. Age was also the only factor that impacts on access, readiness and willingness to engage in telerehabilitation, adding to the literature indicating that the introduction of digital technology such as telehealth, telerehabilitation and telemedicine will increase the digital divide between younger and older patients [26,36]. Interestingly, more females in the study were found to have access to technology as compared to males. This contrasts with an earlier study which showed that digital exclusion remains to be an issue for females worldwide, especially in low- and middle-income countries [37]. Access to technology across the sexes tends to be more equitable in developed countries such as the United Kingdom and the United States [37]. The findings from this study provide further evidence to support the claim that the gender gap is closing in developed high-income countries.

Overall, our results suggest that there is a digital divide between people accessing care in lower socioeconomic areas as compared to the general population. In contrast to the study conducted by Seidman and colleagues in 2017 [25], where 92% of participants had access to technology and 60% were willing to participate in telerehabilitation, our study indicated that use of telerehabilitation may not be as well received by patients receiving care in areas with a lower socioeconomic status or diverse populations. While the provision of technology may help to bridge the access issue, providing adequate support and developing people’s trust in the technology appeared to be more critical for this population [38]. Health service providers also need to reconsider their client/patient demographics before making a decision about redesigning health delivery to cater for our technologically advancing society. A sudden shift to the provision of only telerehabilitation over in-person care may result in greater health disparities by turning people who are currently engaged in health services away. Furthermore, the provision of only telerehabilitation may significantly impact on the health outcomes of older people accessing healthcare, as age appears to be an independent factor influencing access, readiness and willingness to engage in telerehabilitation. This digital divide has been evident during the COVID-19 pandemic, where older people who could not or declined to receive care via telerehabilitation ended up missing out on critical health interventions [26,36,39]. The benchmarking of health services to compare the uptake of telerehabilitation may also not be ideal without taking into account the demographic characteristics of patients residing in the area of service.

A strength of this study is the large sample size included in this prospective study and the ability to accurately captured participant’s CALD status through self-identification [33]. The inclusion of only participants from one local health district may impact the generalisability of this study. Additionally, this study only recruited people who could read and write in at least one of the five selected languages. Considering that the health service provides healthcare for 130 different cultural groups, it is likely that some cultural groups may have been omitted from this study as they read and write in a language other than the selected languages. Despite efforts to provide assistance with reading questions aloud, the study may have also failed to recruit people who were not comfortable with their literacy skills and may have felt uncomfortable with participating in this study. Nonetheless, the socioeconomic demographic is similar to other lower socioeconomic areas in other countries, which suggests that the findings will be applicable to other multicultural populations.

## 5. Conclusions

Being from a CALD background was not an independent predictor influencing access, willingness and readiness to engage in telerehabilitation. Access to technology to engage in allied health telerehabilitation does not mean patients will use telerehabilitation to address their rehabilitation needs. With one in two patients in this study being willing and/or deemed to be telerehabilitation-ready, all people, regardless of their cultural and linguistic abilities, should be given the opportunity to engage with telerehabilitation. Future studies need to look at how health services can provide better support to bridge the growing digital divide in developed countries, especially for older people.

## Figures and Tables

**Table 1:** Participants demographics and telerehabilitation service information.

Variable	CALD *(n* = 145)	No-CALD (*n* = 110)	* Unidentified (*n* = 5)	Overall (*n* = 260)
**Age in years, mean (SD**)	60.4 (15.9)	61.88 (18.2)	60.97 (16.9)	57.4 (19.6)
**Sex** **, *n* (%)**				
Female	93 (64.1)	69 (62.7)	5 (100)	167 (64.2)
Male	51 (35.1)	39 (35.4)		90 (34.6)
Prefer not to say	1 (0.6)	2 (1.8)		3 (1.1)
**Highest level of education completed, *n* (%)**				
Year 8 or below	33 (22.7)	13 (11.8)	1 (20)	47 (18)
Year 10 to Diploma	77 (53.1)	82 (74.5)	4 (80)	163 (62.6)
Bachelor and above	35 (24.1)	15 (13.6)	0	50 (19.2)
**Living with, *n* (%)**				
Alone	16 (11)	24 (21.8)	1 (20)	41 (15.7)
Partner (husband or wife, de facto partner)	41 (28.2)	35 (31.8)	1 (20)	77 (29.6)
Family (Partner and children)	68 (46.8)	41 (37.2)	0	109 (41.9)
Children	18 (12.4)	8 (7.2)	2 (40)	28 (10.7)
Grandchildren	0	0	0	
Sibling	1 (0.6)	0	1 (20)	2 (0.7)
Friend or companion	1 (0.6)	2 (1.8)	0	3 (1.1)
**Employment, *n* (%)**				
Employed	45 (31)	32 (29)	1 (20)	78 (30)
Not employed/others	42 (28.9)	25 (22.7)	2 (40)	69 (26.5)
Retired	22 (15.1)	53 (48.1)	2 (40)	77 (29.6)
**Previous telerehabilitation experiences, *n* (%)**	72 (50.7)	60 (56.6)	1 (20)	132 (53.2)

* Unidentified = Participants did not complete details about their cultural and linguistic backgrounds.

**Table 2:** Characteristic of participants’ access, readiness and willingness to engage in telerehabilitation.

Variable	CALD (*n* = 145)	No-CALD (*n* = 110)	Unidentified (*n* = 5)	Overall (*n* = 260)
**Access, *n* (%)**				
Access	107 (73.7)	75 (68.1)	5 (100)	187 (71.9)
Limited Access	32 (22)	26 (23.6)		58 (22.3)
No Access	5 (3.4)	5 (4.5)		10 (3.8)
Missing	1 (0.6)	4 (3.6)		5 (1.9)
**Device, *n* (%)**				
Smart phone	107 (73.8)	75 (68.1)	4 (80.0)	186 (71.5)
Regular Phone	25 (17.2)	22 (20.0)		47 (18.0)
Shared smart phone/regular phone	7 (4.8)	4 (3.6)		11 (4.2)
Missing	6 (4.1)	9 (8.1)	1 (20.0)	16 (6.1)
**Computer/Internet Skill, *n* (%)**				
Very poor	39 (26.9)	24 (21.8)	1 (20.0)	63 (24.7)
Poor	17 (11.7)	16 (14.5)	1 (20.0)	33 (12.9)
Adequate	32 (22.1)	20 (18.2)	2 (40.0)	52 (20.4)
Good	29 (20.0)	22 (20.0)	1 (20.0)	51 (20.0)
Very good	28 (19.3)	26 (23.6)		54 (21.2)
**Readiness, *n* (%)**				
Yes	54 (37.2)	45 (40.9)	1 (20.0)	99 (38.1)
No	90 (62.1)	61 (55.5)	4 (80.0)	151 (58.1)
Missing	1 (0.7)	4 (3.6)		10 (3.8)
**Willingness to engage in telerehabilitation, *n* (%)**				
Yes	79 (54.4)	58 (52.7)	1 (20.0)	138 (53)
No	55 (37.9)	46 (41.8)	2 (40.0)	103 (39.6)
Missing	11 (7.5)	6 (5.4)	2 (40.0)	19 (7.3)

**Table 3:** Regression analysis between access and demographic characteristics.

Predictors (Reference Variable)	*B* (*SE*)	*p* Value	Odds Ratio	95% CI
**Age**	−0.07 (0.02)	* 0.001	0.94	0.90 to 0.97
**Sex**				
Male (ref)				
Female	0.75 (0.37)	* 0.04	2.11	1.03 to 4.32
**Self-identified cultural background**				
Non-CALD (ref)				
CALD	0.31 (0.38)	0.41	1.36	0.65 to 2.85
**Employment**				
Employed (ref)				
Not-employed/others	−0.57 (0.64)	0.37	0.57	0.16 to 1.98
Retired	−0.92 (0.58)	0.11	0.40	0.13 to 1.24
**Education**				
Year 8 or below (ref)				
Year 10 to Diploma	0.84 (0.44)	0.06	2.30	0.98 to 5.40
Bachelor and above	0.88 (0.67)	0.19	2.41	0.65 to 8.92
**Past Telerehabilitation Experience**				
No (ref)				
Yes	0.58 (0.37)	0.12	1.79	0.87 to 3.68
**Willingness to engage**				
No (ref)				
Yes	1.10 (0.40)	* 0.006	3.00	1.38 to 6.53

* *p* < 0.05, 3 participants who did not identify their sexes were excluded from the analysis.

**Table 4:** Regression analysis between readiness and demographic characteristics.

Predictors (Reference Variable)	*B* (*SE*)	*p* Value	Odds Ratio	95% CI
**Age**	−0.06 (0.15)	* <0.001	0.95	0.92 to 0.98
**Sex**				
Male (ref)				
Female	0.48 (0.39)	0.21	1.62	0.76 to 3.48
**Self-identified cultural background**				
Non-CALD (ref)				
CALD	−0.04 (0.36)	0.90	0.96	0.47 to 1.95
**Employment**				
Employed (ref)				
Not-employed/others	−1.19 (0.49)	* 0.02	0.30	0.12 to 0.79
Retired	−0.85 (0.51)	0.10	0.43	0.16 to 1.16
**Education**				
Year 8 or below (ref)				
Year 10 to Diploma	1.45 (0.68)	* 0.03	4.28	1.12 to 16.34
Bachelor and above	2.25 (0.79)	* 0.005	9.49	2.01 to 44.86
**Past Telerehabilitation Experience**				
No (ref)				
Yes	0.64 (0.36)	0.08	1.90	0.93 to 3.87
**Willingness to engage**				
No (ref)				
Yes	1.21 (0.37)	* <0.001	3.35	1.63 to 6.89

* *p* < 0.05.

**Table 5:** Regression analysis between willingness and demographic characteristics.

Predictors (Reference Variable)	*B* (*SE*)	*p* Value	Odds Ratio	95% CI
**Age**	−0.28 (0.12)	* 0.02	0.97	0.95 to 1.00
**Sex**				
Male (ref)				
Female	0.35 (0.31)	0.26	1.42	0.77 to 2.59
**Self-identified cultural background**				
Non-CALD (ref)				
CALD	−1.4 (0.29)	0.64	0.87	0.49 to 1.54
**Employment**				
Employed (ref)				
Not-employed/others	0.42 (0.41)	0.31	1.51	0.68 to 3.37
Retired	0.37 (0.44)	0.40	1.45	0.61 to 3.47
**Education**				
Year 8 or below (ref)				
Year 10 to Diploma	0.33 (0.41)	0.42	1.39	0.63 to 3.06
Bachelor and above	0.17 (0.53)	0.75	1.19	0.42 to 3.38
**Past Telerehabilitation Experience**				
No (ref)				
Yes	1.00 (0.29)	* <0.001	2.73	1.55 to 4.79

* *p* < 0.05.

## Data Availability

The data presented in this study are available upon request from the corresponding author due to the scope of the ethics approval. Participants did not provide consent to allow other authors to access their personal data.

## Supplementary Materials

The following supporting information can be downloaded at: https://www.mdpi.com/article/10.3390/healthcare12111141/s1, File S1. Study-specific survey.
