# Supplementary material for: Access, Readiness and Willingness to Engage in Allied Health Telerehabilitation Services for Adults: Does Cultural and Linguistic Diversity Make a Difference?
Source: Healthcare (Basel). 2024 Jun 4;12(11):1141. doi: 10.3390/healthcare12111141 (PMC11172156; doi:10.3390/healthcare12111141)
Supplement: Supplementary file 1 [file healthcare-12-01141-s001.zip › healthcare-2962833-supplementary.pdf]

Supplementary Materials File S1. Study-specific survey.

Have you read and understood the attached Participant Information Sheet?

Yes - If yes, proceed to Q2
No – Please read the attached Participant Information sheet and contact the researcher if you have any further questions.

1. I understand that completion of the questionnaire will mean I consent to participate in this study.
    Yes
    No
2. I am happy to complete this questionnaire.
    Yes
    No

Start of survey: About you

1. Age: ______________

2. Sex (please select): ☐Male ☐Female ☐ Other/rather not say

3. Post code: _____________

4. Are you of an Aboriginal or Torres Strait Islander origin? ☐Yes ☐ No

5. Are you an Australian citizen? ☐ Yes ☐ No

If no, what nationality: _____________________________

6. Country of birth: _____________________

7. If you were born overseas, in what year did you first arrive in Australia to live for one year or more? _____________

8. In which country was your mother born?_____________________

9. In which country was your father born? _______________________

10. Do you speak a language other than English at home? ☐Yes ☐ No

11. If yes, what language(s) do you speak at home?

____

12. How well do you speak English?

☐ Very well ☐ Well ☐Not well ☐ Not at all

13. What is your ethnicity? _______________________________________

14. What is your current employment status?

- ☐ Full time
- ☐ Part time/casual
- ☐ Not employed
- ☐ On sick leave
- ☐ Retired
- ☐ Others

15. What is your highest level of education?

- ☐ Did not go to school

- ☐ Year 8 or below （Primary school）
- ☐ Year 10 or equivalent (Early Secondary)
- ☐ Year 11 or equivalent (Secondary)
- ☐ Year 12 or equivalent (High school/college)
- ☐ Diploma
- ☐ Bachelor Degree
- ☐ PhD/Masters Degree

16. Who do you live with at home?

- ☐ Alone
- ☐ Partner (husband or wife, de facto partner)
- ☐ Family (Partner and children)
- ☐ Children
- ☐ Grandchildren
- ☐ Sibling
- ☐ Friend or companion

17. Select one or more of the types of allied health professionals you have you seen over the past year or have an appointment for in the future:

- ☐ Physiotherapy
- ☐ Occupational Therapy
- ☐ Speech Pathology
- ☐ Dietetics
- ☐ Other (please describe)

Access to communication devices

18. Do you have a mobile phone?

- ☐ Yes
- ☐ No

19. What type of mobile phone do you have?

- ☐ Smart phone (e.g.iPhone, Samsung Galaxy)
- ☐ Regular mobile phone with no apps or internet functions (e.g Nokia, LG)
- ☐ Shared smart phone with partner/family member
- ☐ Shared regular phone with partner/family member

20. How often do you use your mobile phone?

- ☐ Daily
- ☐ At least 3-4 times per week
- ☐ Once a week
- ☐ Monthly
- ☐ Less than once per month

☐ Never

21. What do you use your phone for? (Select all the ways you use your way)

☐ Phone calls
☐ SMS
☐ Emailing
☐ Internet searches
☐ Social media (e.g., Facebook, Wechat, Whatsapp)
☐ Apps
☐ Others

22. At home, do you have a computer/ laptop?

☐ Yes
☐ No

23. At home, do you have a Tablet (e.g iPad, Galaxy)?

☐ Yes
☐ No

24. If yes for Q22 and/or Q23, how often do you use these devices?

☐ Daily
☐ At least 3-4 times per week
☐ Once a week
☐ Monthly
☐ Less than once per month
☐ Never

25. Do you use the computer/laptop/tablet to access the internet at home?

☐ Yes
☐ No

26. Do you use a computer, laptop or tablet outside of your home to access the internet?

☐ Yes
☐ No

27. What do you use the computer, laptop or tablet for? (tick all that apply)

☐ Email
☐ Social media (Facebook)
☐ Video calls (Skype, Facetime, Zoom)
☐ Browsing, researching or reading articles on the internet
☐ Online banking
☐ Watch videos or listen to songs (YouTube)
☐ Play games
☐ Shopping

- ☐ Do not use the internet
- ☐ Others

28. Do you have an email address?

- ☐ Yes
- ☐ No

29. If yes for Q28, how often do you read and/or write emails?

- ☐ Daily
- ☐ 3-4 times per week
- ☐ Once per week
- ☐ Monthly
- ☐ Less than once per month
- ☐ Never

30. Overall, how would you rate your computer and internet skills

- ☐ Very poor- ‘I do not access and cannot use the computer or internet independently, or need constant help.’
- ☐ Poor- ‘I need assistance at times.’
- ☐ Adequate- ‘I am able to use the computer and internet within a limited range of use that I need without help.’
- ☐ Good- ‘I am able to use the computer and internet for a wide range of uses with ease without help.’
- ☐ Very good- ‘I am flexible in my navigation of the internet and can use the computer with great confidence.’

31. Will you ever consider receiving telerehabilitation for health if it were available?

- ☐ ‘ I definitely would not use.’
- ☐ ‘I probably would not use.’
- ☐ ‘ I am undecided if I would use it or not’.
- ☐ ‘I probably would use.’
- ☐ ‘I definitely would use.’

32. Have you used telerehabilitation in the past 12 months?

- ☐ Yes
- ☐ No

33. If you said YES to question 32, how have you used telerehabilitation in the past 12-months? (Select all the options that apply)

Type of health appointment	Telephone	Video
GP	☐	☐
Specialist	☐	☐
Physiotherapist	☐	☐
Occupational therapist	☐	☐

Speech Pathologist	☐	☐
Dietician	☐	☐
Other	☐	☐

34. Would you be willing to learn how to access telerehabilitation, if free training and support is available?

- ☐ Yes
- ☐ No

Please return your questionnaire to the researcher at your clinic today, or give it to your clinician, or return it by post in the reply-paid envelope. Remember, do not write your name on this questionnaire or the envelope, so your responses remain anonymous.
